# Predicting return to work after acute myocardial infarction: Socio-occupational factors overcome clinical conditions

**DOI:** 10.1371/journal.pone.0208842

**Published:** 2018-12-13

**Authors:** Mariarita Stendardo, Melissa Bonci, Valeria Casillo, Rossella Miglio, Giulia Giovannini, Marco Nardini, Gianluca Campo, Alessandro Fucili, Piera Boschetto

**Affiliations:** 1 Department of Medical Sciences, University of Ferrara, Ferrara, Italy; 2 Department of Statistical Sciences "Paolo Fortunati", University of Bologna, Bologna, Italy; 3 Department of Prevention and Protection, University-Hospital and Public Health Service of Ferrara, Ferrara, Italy; 4 Cardiology Unit, University-Hospital of Ferrara, Cona, Ferrara and Maria Cecilia Hospital, GVM Care & Research, E.S: Health Science Foundation, Cotignola, Ravenna, Italy; Azienda Ospedaliero Universitaria Careggi, ITALY

## Abstract

**Objectives:**

Return to work after acute myocardial infarction (AMI), a leading cause of death globally, is a multidimensional process influenced by clinical, psychological, social and occupational factors, the single impact of which, however, is still not well defined. The objective of this study was to investigate these 4 factors on return to work (RTW) within 365 days after AMI in a homogeneous cohort of patients who had undergone an urgent coronary angioplasty.

**Participants:**

We studied 102 patients, in employment at the time of AMI (88.24% of men), admitted to the Department of Cardiology of the University-Hospital of Ferrara between March 2015 to December 2016. Demographical and clinical characteristics were obtained from the cardiological records. After completing an interview on social and occupational variables and the Hospital Anxiety and Depression (HADS) questionnaire, patients underwent exercise capacity measurement and spirometry.

**Results:**

Of the 102 patients, only 12 (12.76%) held a university degree, 68.63% were employees and 31.37% self-employed. The median number of sick-leave days was 44 (IQR 33–88). At day 30, 78.5% of all subjects had not returned to work, at day 60, 40.8% and at day 365 only 7.3% had not resumed working. At univariate analyses, educational degree (p = 0.026), self-employment status (p = 0.0005), white collar professional category (p = 0.020) and HADS depression score were significant for earlier return to work. The multivariate analysis confirms that having a university degree, being self-employed and presenting a lower value of HADS depression score increase the probability of a quicker return to work.

**Conclusions:**

These findings suggest that the strongest predictors of returning to work within 1 year after discharge for an acute myocardial infarction are related more to socio-occupational than to clinical parameters.

## Introduction

Coronary heart disease (CHD) is the leading cause of mortality and morbidity in industrialized countries and acute myocardial infarction (AMI) is one of the five main manifestations of CHD.

Almost 45% of patients affected by myocardial infarction are of working age [1], in Italy defined as those aged 18 to 65, and this percentage is expected to increase with the aging of the working population. Although infarction mortality is high, the introduction of new treatment regimens for acute management and primary and secondary prevention have improved the prognosis [2]. This has led to an increasing number of survivors returning to work after treatment [3]. Even though the main standards for the quality of care are fewer post-AMI complications such as recurrent AMI, heart failure and death, returning to work also deserves to be considered an important marker of functional status and a significant component of individual self-esteem and social costs [2]. Generally, employment is related to higher health and greater social welfare, whereas jobless produces negative effects on the subject’s physical and mental health as well as financial hardship due to loss of income [4]. Return to work is a significant part of complete recovery and successful social reintegration after AMI. There are considerable differences between countries in timing and rates of return to work: the median time is 50 days and the rates of return to work within 1 year vary between 60% and 93% [2]. Indeed, return to work is a complex process determined by the composite interplay of physical, psychological, socio-demographic and occupational factors. However, the impact of these factors on resumption of paid employment is controversial as some studies indicate physical elements, some the psychological and others the socio-demographic or the occupational [1,5–7]. Discrepancies in this multitude of factors may be due to non-homogeneous study populations with regard to the performed invasive coronary procedures [percutaneous coronary intervention (PCI) or coronary artery bypass grafting] and whether or not to undergo cardiac rehabilitation. Other possible causes of the inconsistent results in available literature may be: the different study design, in some investigations retrospective, in others prospective and the omitted evaluation of one or more of the main factor groups (i.e. physical, psychological, socio-demographic and occupational) [8,9].

The aim of this study was, therefore, to investigate the impact of physical, psychological, socio-demographic and occupational factors on return to work within 1 year after AMI in a homogeneous cohort of patients who had undergone coronary angioplasty and did not have post-operative rehabilitative physical therapy as it is not provided by our University-Hospital.

## Materials and methods

### Study design and subjects

The study was approved by the Ethics Committee of Ferrara, Italy. Approval number: 150387. A written informed consent was obtained from all participants.

From March 2015 to December 2016 a prospective cohort study was carried out. We enrolled 102 consecutive patients admitted to the Department of Cardiology of the University-Hospital of Ferrara for acute myocardial infarction, diagnosed according to the criteria of the European Cardiac Society [10]. Only patients treated with percutaneous coronary intervention and those in employment at the time of the cardiac event were included in the study. None of the patients performed rehabilitative physical therapy post-AMI as it is not included in our University-Hospital treatment plan. For every subject we collected: 1. demographic data: age, gender, cohabitation (living with a partner or single) and educational level (primary/secondary school, high school or university degree), 2. smoking history (smokers/ex-smokers or never smokers) and the number of pack-years of cigarettes smoked, 3. medical and cardiac history. We also registered employment status at the time of AMI (using the term employee when a worker is employed by another or the term self-employed) and professional category (white collar worker defines who performs semi-professional office, administrative and sales-coordination task as opposed to blue collar worker whose job requires manual labor). Type of the infarction [ST-elevation myocardial infarction (STEMI) and non-ST-elevation myocardial infarction (NSTEMI)], left ventricle ejection fraction (LVEF) from echocardiography performed prior to hospital discharge and comorbidities from personal medical records were noted.

At one month visit (from the AMI), patients were asked for cardio-respiratory symptoms, chest pain, dyspnea, palpitations and syncope, and the date returned to work (where applicable). Return to work (RTW) is defined as employment status resumption. Anxious and depressive symptoms were assessed using the Italian version of the Hospital Anxiety and Depression Scale (HADS) [11]. Exercise capacity was expressed in terms of metabolic equivalents (METs) and measured by peak oxygen consumption (VO_2peak_) using both the six-minute walk test (6MWT) and 30-second chair stand test (30SCS); pulmonary function was evaluated by spirometry. All patients were followed up until one year after the AMI to investigate their return to work. The study was approved by the local Ethics Committee and performed in accordance with the ethical standards laid down in the 1964 Declaration of Helsinki and its later amendments. A written informed consent was obtained from all participants after being informed by a physician on the rationale and aims of the survey (ClinicalTrials.gov number, N. 150387).

### Assessment of exercise capacity

To assess VO_2peak_, we performed 6 minute walk test and 30 second chair stand test. In particular, VO2 peak was measured through the equation developed by Mandic et al., based on 6MWT distance and combination of demographic (age, gender), anthropometric (height, weight, body mass index) and functional variables (30-second chair stand test) [12]. METs were computed by taking the energy costs (VO2 peak ml·kg-1·min-1) and dividing them by 3.5 ml·kg-1·min-1 [13].

### Six-minute walk test

The 6MWT was performed according to American Thoracic Society guidelines for adults [14]. Briefly, the 6MWT is a self-paced test of functional exercise capacity in which patients are asked to walk as far as possible in 6 minutes along a flat corridor. After 6 minutes, patients are instructed to stop walking and the distance covered is recorded.

### 30-second chair stand test

30SCS was administered using a chair without armrests. The chair was placed against a wall to prevent it from moving. The participants were encouraged to complete as many full stands as possible within 30 seconds and they were instructed to fully sit between each stand [15].

### Hospital anxiety and depression scale

HADS is a 14-item scale with responses scored 0–3 (3 indicating higher symptom frequencies). The score for each subscale (anxiety and depression) ranged 0–21, with scores categorized as follows: normal (0–7), mild (8–10), moderate (11–14), and severe (15–21)[16].

### Spirometry

Forced expiratory volume (FEV_1_), forced vital capacity (FVC) and the FEV_1_/FVC ratio were measured using a spirometer (Biomedin, Padova, Italy). The best of three values was expressed as a percentage of the predicted normal value. All measurements were obtained and interpreted in accordance with the recommendations of the American Thoracic Society/European Respiratory Society [17].

### Statistical analysis

Qualitative variables were presented as frequencies and percentages. When quantitative variables were normally distributed, the results were expressed as mean values and standard deviation (SD), otherwise median and interquartile range (IQR; 25–75th percentile).

Survival analyses were performed to study predictors of RTW after an acute myocardial infarction. Kaplan–Meier curves were estimated over the 12 month follow up period.

To determine predictors of time for return to work, the log-rank test was used to detect differences in time required across categorical variables, univariate analysis was performed for quantitative variables and multivariate Cox regression analysis was applied to confirm the significance of the socio-occupational and clinical predictors after adjusting for confounders. In the multivariate model the variables that resulted significant at the univariate analyses and those close to significance and clinically meaningful were included. In this model the dependent variable was the timing to return to work status and the independent variables were high school, university degree, METs physical performance, self-employed and HADS-D score. The hazard ratios (HR) were reported with 95% confidence interval (CI). Proportional hazards assumption was assessed using tests based on the Schoenfeld partial residuals [18,19].

Data analysis was performed with STATA/IC statistical package (release 12.0, Stata Corporation) and a p-value lower than 0.05 was considered statistically significant.

## Results

### General characteristics of the study population

Baseline socio-demographic, clinical and occupational characteristics of the 102 patients who had undergone a coronary angioplasty due to AMI are presented in Table 1.

**Table 1:** Baseline characteristics of study patients.

**Men, No. (%)**	90 (88.24)
**BMI (Kg/m2)**	27.1 [24.84–29.70]
**Smoking history, No. (%)**	
• Smokers/ex-smokers	78 (76.47)
• Never smokers	24 (23.53)
**Pack/years**	20 [5–39]
**Systolic pressure**	122.25 ± 11.76
**Diastolic pressure**	76.37 ± 8.29
**Type of AMI, No. (%)**	
• STEMI	64 (62.75)
• NSTEMI	38 (37.25)
**Left ventricular ejection fraction (%)**	50.22 ± 8.16
**Cohabitation, No. (%)**	
• Living with a partner	83 (86.46)
• Single	13 (13.54)
**Educational level, No. (%)**	
• Primary/secondary school	41 (43.62)
• High school	41 (43.62)
• University degree	12 (12.76)
**Employment status, No. (%)**	
• Employee	70 (68.63)
• Self-employed	32 (31.37)
**Professional category, No. (%)**	
• White collar worker	48 (47.06)
• Blue collar worker	54 (52.94)

Data are expressed as mean ± standard deviation or median [interquartile ranges: 25th-75th percentile] or number of subjects (%). BMI: body mass index; AMI: acute myocardial infarction; STEMI: ST-elevation myocardial infarction; NSTEMI: non-ST-elevation myocardial infarction.

The median age of the total sample was 56 (interquartile ranges 25th-75th percentile IQR 50–60). The majority of the patients were male (88.24%) and were employees (68.63%). While white and blue-collar workers were equally distributed in the study population (47.06% vs 52.94%), only 12 (12.76%) subjects held a university degree. In 32.35% of the study participants, AMI was associated with other diseases (comorbidities) of which the most prevalent were hypertension (57%), diabetes (15%) and depression (7%).

At 1 month follow-up visit (Table 2), sixty-one subjects (59.80%) did not refer cardiac symptoms since their discharge, the remaining patients complained of dyspnea (27%), palpitations (12%), chest pain (8%) and syncope (5%). The median estimated MET values of patient exercise capacity was 6.03 (IQR 5.50–6.53). Both the HADS questionnaire scores for anxiety/depression and spirometry parameters (FEV_1_, FVC and FEV_1_/FVC) were within the normal reference values. The median length of hospitalization was 4 (IQR 4–6) days.

**Table 2:** Functional and clinical characteristics of study patients at one month follow-up visit.

Variables	Total
	(n = 102)
**Cardiac symptoms, No. (%)**	
Yes	41 (40.20)
• dyspnea	28 (27)
• palpitations	12 (12)
• chest pain	8 (8)
• syncope	5 (5)
No	61 (59.80)
**Spirometry**	
• FEV1% predicted	103.31±15.53
• FVC % predicted	107 [96.5–118]
• FEV1/FVC (%)	78 [73.25–81]
**METs Physical Performance**	6.03 [5.50–6.53]
**HADS-A score**	3 [1–6]
**HADS-D score**	2 [1–5]
**Days of hospitalization**	4 [4–6]
**Sickness absence days**	44 [33–88]

Data are expressed as mean ± standard deviation or median [interquartile ranges: 25th-75th percentile] or number of subjects (%). FEV1, forced expiratory volume in 1 second; FVC, forced vital capacity; HADS-A score: score of anxiety subscale of the Hospital Anxiety and Depression Scale; HADS-D score: score of depression subscale of the Hospital Anxiety and Depression Scale; MET: metabolic equivalent.

### Predictors of time to return to work

**Fig 1:**
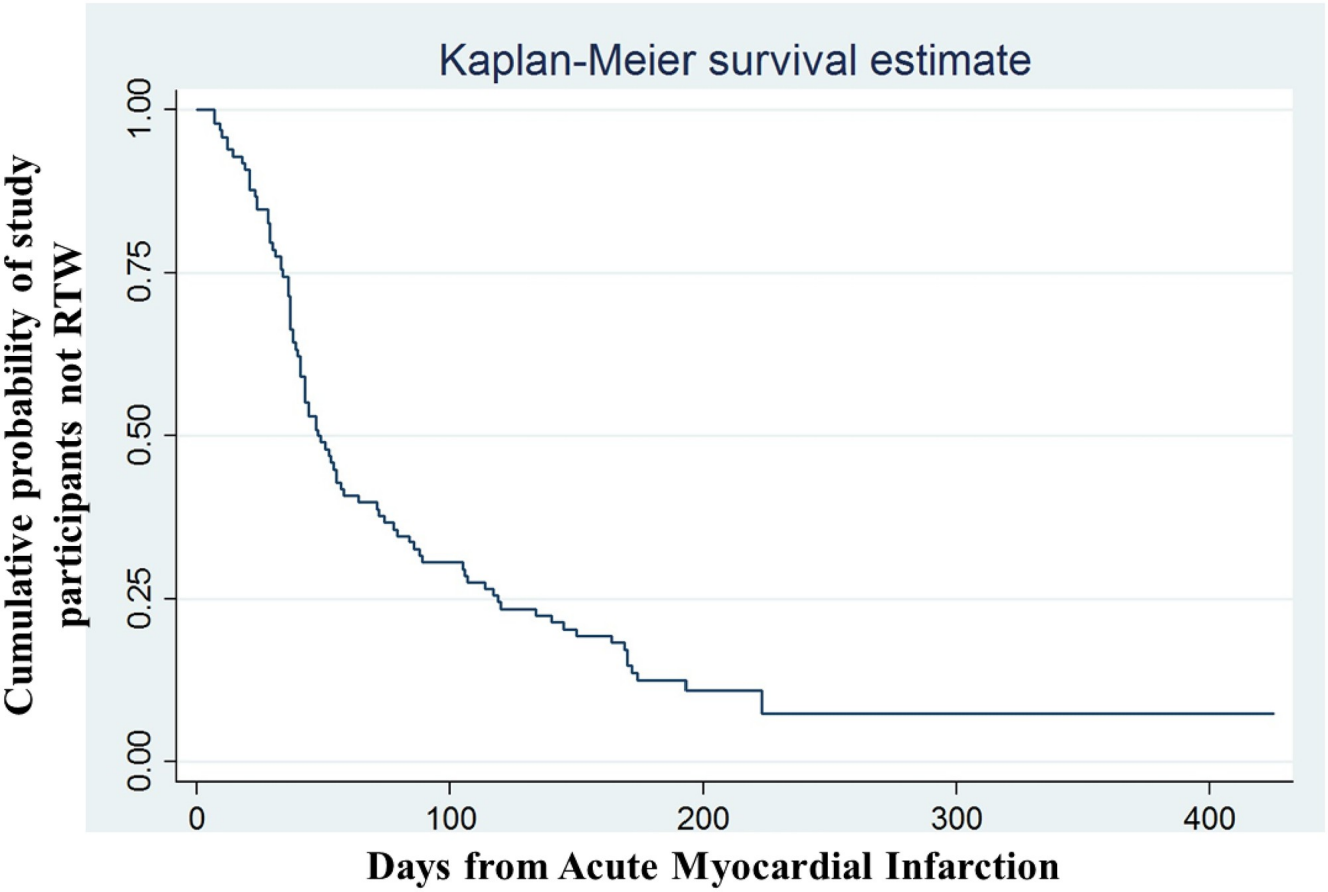
Rate of no return to work status (RTW) in the study patients during the 12 month follow-up.

The cumulative probability of study participants of no return to work, analyzed by the Kaplan–Meier survival estimate as a function of time (days) elapsed from AMI, is shown in Fig 1.

At day 30, 78.5% of all subjects had not returned to work; at day 60, 40.8% and at day 365 only 7.3% had not resumed working. Of these 7 subjects: 4 had finished their short-term employment contracts, 1 had had a second AMI, 1 decided to leave his very stressful job and in one case the employer closed the business. The median time of sick-leave was 44 days (IQR 33–88).

The qualitative variables, shown in Table 3, emerged as significant for earlier return to work with log-rank test were: educational degree (p = 0.026), self-employment status (p = 0.0005) and white collar professional category (p = 0.020).

**Table 3:** Log-rank test of the association between timing of return to work status (RTW) and qualitative socio-demographic, clinical and occupational variables.

RTW	p-value
**Gender**	0.741
**Cohabitation status**	0.634
**Educational level**	**0.026**
**Type of AMI**	0.504
Cardiac symptoms*	**0.105**
**Self employed**	**<0.001**
**White collar worker**	**0.020**

AMI: acute myocardial infarction

*absence/presence of at least one symptom

Table 4 presents the data of the univariate Cox regression analysis for quantitative predictors of return to work; only HADS scores were associated with early RTW.

**Table 4:** Univariate Cox regression of the association between timing of return to work status (RTW) and quantitative clinical, functional, psychological and socio-demographic variables.

RTW	P-value	HR	95% CI
**Age (years)**	0.213	1.020	0.988–1.054
**BMI (Kg/m2)**	0.848	1.005	0.950–1.064
**Pack-years**	0.789	0.998	0.988–1.010
**Systolic pressure**	0.927	0.999	0.982–1.016
**Diastolic pressure**	0.426	0.989	0.965–1.015
**Left ventricular ejection fraction (%)**	0.495	1.009	0.983–1.035
**METs Physical Performance**	0.135	1.173	0.951–1.447
**Days of hospitalization**	0.178	0.930	0.837–1.033
**HADS-A score**	**0.009**	0.913	0.852–0.978
**HADS-D score**	**0.008**	0.899	0.831–0.972
**FEV1% predicted**	0.522	1.004	0.991–1.017
**FVC % predicted**	0.794	1.001	0.989–1.014
**FEV1/FVC (%)**	0.709	1.006	0.973–1.039

HR: Hazard Ratio, CI: Confidence Interval

BMI: body mass index; MET: metabolic equivalent; HADS-A score: score of anxiety subscale of the Hospital Anxiety and Depression Scale; HADS-D score: score of depression subscale of the Hospital Anxiety and Depression Scale; FEV1, forced expiratory volume in 1 second; FVC, forced vital capacity.

The multivariate analysis confirms that having a university degree, being self-employed and presenting a lower value of HADS-D score increase the probability of an earlier return to work (Table 5).

**Table 5:** Multivariate Cox regression analysis for predictors of return to work status (RTW).

RTW	P-value	HR	95% CI
**High school**	0.763	1.080	0.655–1.782
**University degree**	**0.002**	3.397	1.574–7.330
**METs Physical Performance**	0.447	1.091	0.871–1.367
**Self employed**	**<0.001**	2.647	1.560–4.490
**HADS-D score**	**0.038**	0.910	0.832–0.995

HR: Hazard Ratio, CI: Confidence Interval

MET: metabolic equivalent; HADS-D score: score of depression subscale of the Hospital Anxiety and Depression Scale.

## Discussion

This study investigates the return to work in post-AMI patients and it has been found that about 7% of the subjects did not return to work within 1 year. Earlier RTW was influenced more by the patient’s socio-occupational factors than his clinical conditions. Indeed, the predictors of an earlier return to work were: self-employment status, higher educational level (i.e. having a university degree) and lower, but still within the normal range, values of HADS depression score (i.e. a good mood).

Compared to most previous literature, our results showed a considerably lower proportion of subjects who did not return to work within 1 year after AMI [20–22], but are in line with those published recently by Warraich et al. In agreement with these authors we hypothesize that advancement in AMI care (improvement of treatments and reduction of mortality), which has led to a better functional recovery of patients, could have promoted their return to work [23].

In our study the most important factor associated with an earlier RTW is the self-employment status. Since 1985 there has been evidence that RTW is more frequent in the self-employed than in the employees [24] and a recent Swedish study reported that being self-employed was associated with a lower risk of long-term sickness absence following a coronary revascularization [25]. Our results, largely supported by previous data, might be explained primarily by the fact that the self-employed feel they cannot afford to stay on long-term sick leave as Italy does not provide them with statutory sick pay. Furthermore they can adapt their work situation to suit their clinical condition.

The finding of the relationship between a higher education level and an earlier work resumption has already been reported. A number of studies have examined the association between different indicators of socioeconomic position (such as education, income and social class) and return to work after AMI. Our results are in line with those of Smedegaard et al. who performed a nationwide Danish retrospective study in a cohort of patients discharged after AMI and found that income and high education level encouraged maintenance of employment [2]. Other preceding studies showed that white collar workers were more likely to RTW than blue collar workers employed in low income jobs with lower decision latitudes, a known strong stressor and factor associated with no return to work after a cardiac event [26]. To our knowledge this is the first study that suggests, by prospectively analyzing the level of education as a possible predictor of return to work, that only patients with a university degree are more likely to resume work earlier.

Concerning the association between functional capacity and return to work, we found that the physical performance of patients evaluated by MET was not associated with an earlier RTW. High exercise capacity on the exercise tests has been connected with work resumption [27], but evidence on its occupational performance impact is inconsistent throughout previous literature. Indeed, in a survey of 90 patients who had suffered an acute myocardial infarction and were evaluated after 12 months in general practice it was shown that exercise tests could not predict the chances of returning to work [28]. As mentioned above, also the nature of the job (manual or clerical) may influence return to work post-AMI, manual work being a possible cause of delay [29]. In the unadjusted analysis we found a significant correlation between clerical work and early RTW, but this was not confirmed when the model was adjusted for confounders. Functional capacity is linked to the ability to carry out a manual job and a recent report has highlighted that manual or semiskilled work (which may require intense physical activity) was associated with a lower probability of returning to work after one year [4]. In our study population it was consistently observed that neither physical performance nor manual work were independent predictors of work resumption. Taken together, these data indicate that the association between physical job demand, exercise performance and return to work is rather complex and may deserve further investigation.

Depression is common after MI and the condition of working less or not working at 1-year post-AMI is associated with higher rates of depression [23]. Depression documented between hospitalization and few months after discharge can predict delay or failure in RTW after a cardiac event [5]. Although depressive disorder is frequent after a myocardial infarction, it often remains neglected in this population. This may be due to the brief hospitalization required for a MI (the average length of hospital stay is now 3–5 days) and to the symptoms overlap between these two diseases. In our study a higher score of HADS depression subscale, but still within the normal range, decreases the likelihood of returning to work early. As far as we know, this is the first time that mood decline rather than a proper depressive disorder is associated with return to work.

Strengths of our study are: 1) to have accurately analyzed a population homogeneous with regard to therapeutic treatment (all first-time PCI-treated patients without post-operative rehabilitative physical therapy) and 2) to have examined simultaneously the impact of the four types of the predictors of work resumption after AMI i.e. the clinical, psychological, social and occupational. Nevertheless, our study also has some limitations. The sample size was relatively small and recruited from a single hospital, thus results cannot be considered as representative of the general population and should be interpreted with caution. A specific evaluation of work physical request was not performed and working conditions were classified according only to the nature of the job (manual or clerical). None of our patients underwent post-operative rehabilitative physical therapy precluding evaluation of its impact on timing to resume work. However, most of the hospitals in Italy do not provide a rehabilitation program to AMI patients treated with angioplasty. The date of return to work and therefore the numbers of sickness absence days are patient self-reported and do not arise from the consultation of appropriate registers. Given the observational nature of this study we cannot make inferences on causality when examining outcomes. Lastly, the gender distribution of the present cohort was not balanced, women being under-represented as they tend to suffer from AMI at an older age compared with males [30].

In conclusion, this study shows that the strongest predictors of return to work within 1-year after discharge for an acute myocardial infarction treated with percutaneous coronary intervention are: self-employment status, higher educational level and a good mood. Our results suggest the need of evaluating psychosocial conditions of patients after AMI and the probable advantage of interventions such as psychological therapy. However, more research is deserved to improve timing of resuming work after an acute myocardial infarction.

## Supporting information

S1 File Relevant data for the statistical analysis. (XLSX)
